# BCL7A as a novel prognostic biomarker for glioma patients

**DOI:** 10.1186/s12967-021-03003-0

**Published:** 2021-08-06

**Authors:** Junhui Liu, Lun Gao, Baowei Ji, Rongxin Geng, Jing Chen, Xiang Tao, Qiang Cai, Zhibiao Chen

**Affiliations:** 1 Department of Neurosurgery, Renmin Hospital of Wuhan University, No.238, Jiefang Road, Wuchang District, Wuhan, 430060 Hubei China; 2 Central Laboratory, Renmin Hospital of Wuhan University, No.238, Jiefang Road, Wuchang District, Wuhan, 430060 Hubei China

**Keywords:** BCL7 family, Glioma, Prognosis, Immune, Temozolomide

## Abstract

**Background:**

Glioma is the most common primary brain tumor and represents one of the most aggressive and lethal types of human cancer. BCL7 family has been found in several cancer types and could be involved in tumor progression. While the role of BCL7 family in human glioma has remained to be elucidated.

**Methods:**

Paraffin-embedded tumor samples were obtained to detect BCL7 expression by performing in glioma. Data (including normalized gene expression and corresponding clinical data) were obtained from Gliovis, CGGA, GEO, cBioportal and Oncomine and were used to investigate BCL7 genes expression in glioma. Survival analyses were calculated by Kaplan–Meier methods and Cox regression analysis in TCGA and CGGA. Gene Set Enrichment Analyses (GSEA) and gene ontology (GO) analysis was employed to perform the biological processes enrichment.

**Results:**

BCL7A expression in glioma tissues was lower compared to non-tumor brain tissues (NBT), and exhibited a negative correlation with glioma grades. Results from immunohistochemical (IHC) staining and public dataset validation demonstrated that BCL7B and BCL7C were highly expressed in glioma tissues compared to NBT. Cox regression analysis identified BCL7A as the only gene in the BCL7 family that was independently associated with the prognosis of lower-grade glioma (LGG) and glioblastoma (GBM). GO and GSEA analyses revealed the potential contribution of BCL7A in adaptive immune response and neutrophil activation in the tumor microenvironment. Moreover, we found that BCL7A had no prognostic effect on the overall survival of GBM patients who received IR only; however, patients who received chemotherapy (TMZ) combined with IR in the high BCL7A group survived longer than patients in the low BCL7A group (HR = 0.346, p < 0.05).

**Conclusion:**

BCL7A is a new tumor suppressor gene and can be adopted as a biomarker for independent prognosis in glioma and to evaluate response to TMZ.

## Introduction

Gliomas account for nearly 80% of primary malignant brain tumors [1]. According to the World Health Organization (WHO) classification, gliomas occur in four grades. Notably, glioblastoma (GBM, WHO IV) register 56.6% of all cases and has fetal clinical prognosis. Following diagnosis reports between 2000 and 2014, the 1-year and 5-year relative survival rates (RS) of GBM patients were 41.4% and 5.4%, respectively [2]. The prognosis of low-grade glioma (LGG) patients is much better than GBM patients; however, a majority of LGGs patients eventually progress to high-grade gliomas (HGG) [3, 4]. Although the application of the STUPP regimen has improved the prognosis of patients with GBM, the effects remain far from satisfactory. Molecular biomarkers, such as IDH, 1p19q, MGMT promoter methylation, and ATRX, are vital in predicting tumor malignancy and clinical prognosis. Such molecular alterations are crucial in understanding the classification, diagnosis, and management of gliomas. This calls for urgent exploration to identify more novel molecular markers to predict prognosis and guide treatment.

B-cell leukemia protein 7 (BCL7) protein family is identified as the subunit of the Switch/Sucrose Non-Fermenting (SWI/SNF) complex, one of the ATP-dependent chromatin remodelers [5, 6]. Located in chromosomes 12 and 16, the BCL7 gene family has a functional domain containing a conserved amino-terminal region. A previous study implicated that this protein family participates in chromatin remodeling owing to their interaction with SWI/SNF components [7]. Other reports have revealed the association of the BCL7 gene family with various tumors. BCL7A, a member of the BCL7 family, was identified as a risk factor associated with Burkitt lymphoma [8], non-Hodgkin’s lymphoma [9], and cutaneous T cell lymphoma [10]. In B cell malignancy, BCL7A was overexpressed in late B cell differentiation. Contrarily, it was lowly expressed in mature T cells and late plasma cells. BCL7A protein was mainly expressed in precursor and mature B cell lymphomas, especially in germinal center (GC)-related lymphomas [11]. A recent study by Uehara et al. found that BCL7B was essential for the self-renew and differentiation of epithelial seam cells, it exerted a crucial role in regulating the Wnt-signaling pathway and the apoptotic pathway [12]. Pancreatic cancer patients expressing high BCL7B levels had a better clinical prognosis than those expressing low BCL7B levels. Of note, inhibition of BCL7B in pancreatic cancer cells could dramatically reduce the invasion and motility of cancer cells by regulating CREB signaling [13]. Researchers need to explore the role of the BCL7 gene family in glioma, particularly on their expression pattern and potentially associated genetic functions.

In this study, we employed public datasets, including the Cancer Genome Atlas-GBMLGG, Chinese Glioma Genome Atlas-mRNAseq_693#, Rembrandt, and GSE16011 to explore the expression level of the BCL7 family and their correlation with clinicopathological variables and prognosis. Further validation of the expressions of the BCL7 family in glioma was undertaken via IHC staining of 108 paraffin-embedded glioma samples. The present study comprehensively screened for BCL7 genes associated with malignancy in glioma, explored their biological role, and evaluated how their expression is associated with prognosis.

## Methods

### Glioma tissues

We used a paraffin-embedded glioma tissue microarray (108 glioma samples and 10 non-tumor brain tissues). All specimens were acquired from hospitalized patients between January 2016 and March 2018 in the department of neurosurgery of Renmin Hospital of Wuhan University. Of the patients, none received any chemo- or radiotherapy before surgery. All patients signed informed consent. Approval for this study was issued by the Institutional Ethics Committee of the Faculty of Medicine at Renmin Hospital of Wuhan University [approval number: 2012LKSZ (010) H].

### Immunohistochemical (IHC) staining

The paraffin-embedded tissue microarray was heated in an oven (60 °C) for 90 min. Then, slides were placed in xylene (3 × 5 min/time) and ethanol at varying concentrations (100%, 95%, and 75%) for hydration treatment. After three times PBS wash, 3% H_2_O_2_ was added to slides and left for 10 min at room temperature. Slides were then completely immersed in the antigen retrieval liquid at 95°C for 10 min and allowed to cool naturally. Triton-PBS (100X) was used for 5 min and the slides were blocked with 1% BSA for 30 min. After that, primary antibodies were added, followed by overnight incubation at 4 °C. The next day, the slides were washed with PBS (3 × 10 min/time), and then incubated with HRP goat anti-rabbit/mouse IgG for 1 h. Thereafter, DAB was added dropwise to the slides, and the color reaction was stopped using tap water. Hematoxylin staining was repeatedly performed for 1 min, and the color was separated with 1% hydrochloric acid alcohol solution. Finally, slides were covered with neutral balsam and with an Olympus BX40 microscope (Tokyo, Japan), images were acquired.

### IHC evaluation

Here, both intensity and the percentage of immune-reactive cells were evaluated. Positive staining rate was scored as: 0 points for less than 5%; 1 point for 5–25%; 2 points for 26–50%; 3 points for 51–75%; 4 points for 75%. Notably, the scores 0, 1, 2, and 3 denoted no staining, weak staining, moderate staining, and strong staining, respectively. The two scores were multiplied to get the final score. IHC scores between 0–4 and 5–12 represented low protein expression and high protein expression, respectively. Two individuals independently assessed the IHC staining results.

### Oncomine and cBioportal

The mRNA expression of BCL7 genes in glioblastoma was explored via the oncomine platform (www.oncomine.org) [14]. p-value less than 0.05 denoted statistical significance. The genomic alterations of the BCL7 family in TCGA-GBM were identified in the cBioPortal platform (http://www.cbioportal.org/) [15].

### Gene Expression Omnibus (GEO)

A public dataset, GEO (https://www.ncbi.nlm.nih.gov/geo/), was employed to assess the expression of the BCL7 family in glioma. Normalized RNAseq expression and corresponding clinical information of GSE42456, GSE44971, GSE50165, GSE66357 were used in differential expression analysis.

### Gliovis analysis

Gliovis website (http://gliovis.bioinfo.cnio.es/), an important platform was adopted for data visualization and analysis to explore brain tumors [16]. In addition to the normalized gene expression data, the website includes information on glioma molecular pathology and glioma subtypes, these are crucial tools for online analysis of glioma.

### Gene Set Enrichment Analyses (GSEA)

The expression profiles of GSE16011 (Gravendeel) were downloaded from the GEO data base. GSEA (http://software.broadinstitute.org/gsea/index.jsp) was employed to analyze potential genes associated with BCL7A. According to the real hub genes (top 50% vs bottom 50%), they were separated into high and low groups. GSEA was then used for biological processes enrichment [17]. GSEA enrichments were estimated using the normalized enrichment score (NES). The significance of the enrichment was assessed at FDR < 0.25 level, p-value < 0.05, and FDR < 0.25 levels.

### Statistical analysis

Data were expressed as mean ± standard deviation (SD) or ± standard error of the mean. Significant differences between the means ± the standard deviation of two different groups were examined using a Students’ t-test; notably, one-way ANOVA was used for more than two groups. Spearman correlation analysis was used to examine the correlation analysis. Patients were divided into high and low groups according to the 50% cutoff point of gene expression. Differences in survival between groups were evaluated via Kaplan–Meier Survival analysis with a log-rank significance test. Univariate and multivariate Cox regression models were applied to detect the prognostic elements. GraphPad Prism 5.0 software (GraphPad Inc., San Diego, CA, USA) was used to generate the graphs.

## Results

### Transcriptional level of BCL7s in glioma

**Fig. 1:**
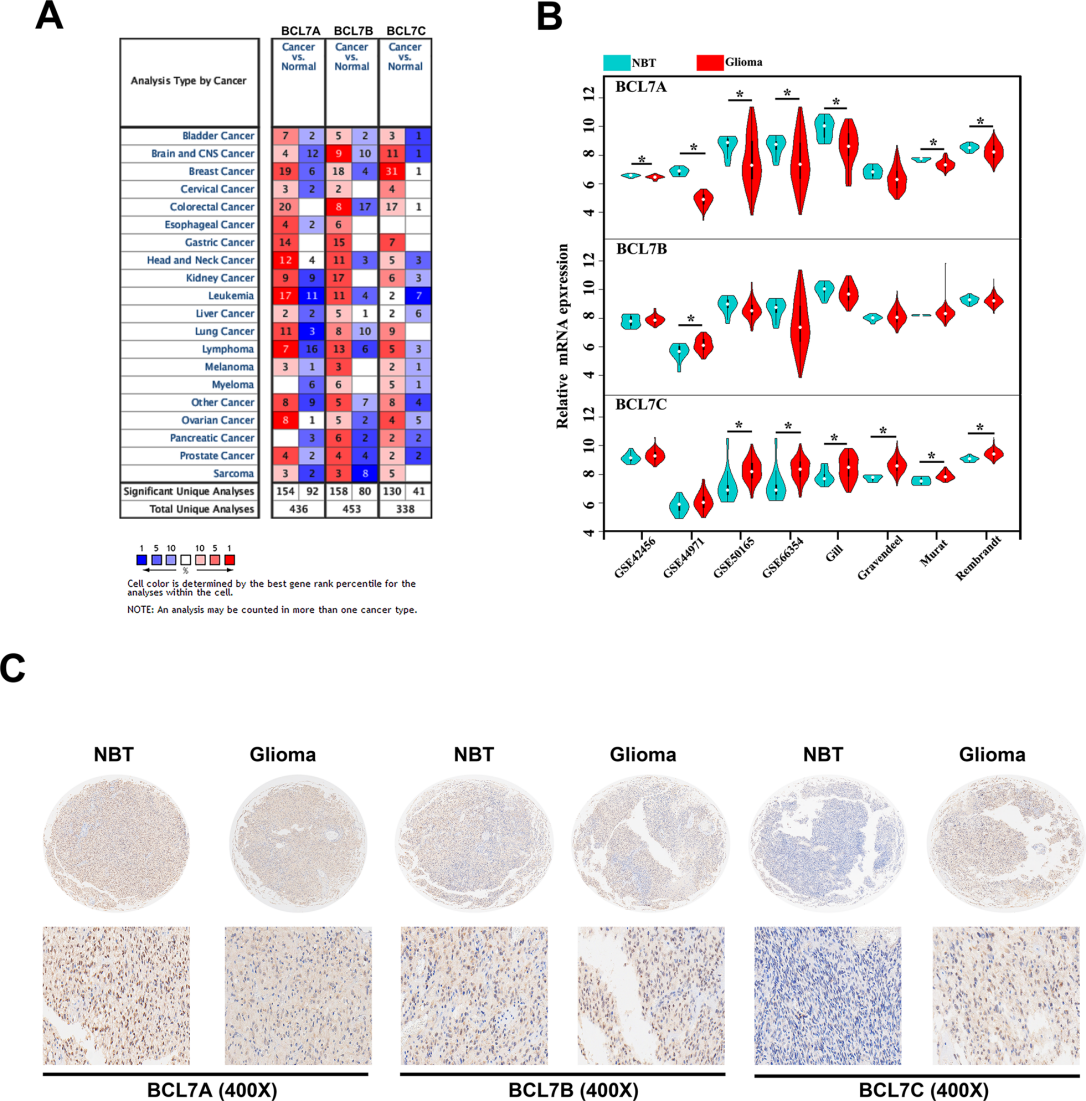
Transcriptional level of BCL7s in glioma. **A** The expression of BCL7 family in different types of cancers in Oncomine. **B** Eight public datasets contained mRNA data of glioma tissues and matched non-tumor brain tissues downloaded from Gliovis and GEO datasets. **C** Immunohistochemical (IHC) staining of BCL7 genes in 108 glioma tissues and 10 non-tumor brain tissues. p < 0.05. NBT, non-tumor brain tissues

To acquire more comprehensive information on the expression of the BCL7s family, we employed the cBioportal, Oncomine, Gliovis, and GEO datasets to assess for gene expression for BCL7s family in glioma. We found low BCL7A expression in 12 of 16 studies and high BCL7C expression in 11 of 12 studies in brain and CNS cancer (Fig. 1A). To further illustrate the expression of BCL7 genes in glioma, we used multiple datasets from GEO. BCL7A mRNA expression was significantly low in glioma tissues compared to normal brain tissues (NBTs) in 7 out of the 8 datasets, except for the Gravendeel dataset (Fig. 1B). In GSE44791, whilst BCL7B was highly expressed in glioma compared to NBT, we found no significant differences for the other seven datasets (Fig. 1B). Remarkably, there was evidence that BCL7C expression was highly elevated in glioma in 6 out of 8 public datasets. However, BCL7C expression in glioma did not differ from that of NBTs in GSE42456 and GSE44971 datasets (Fig. 1B). To verify these findings, IHC was performed to detect BCL7 gene expression in 108 glioma tissues. Notably, the expression of BCL7A and BCL7C was significantly lower and higher in glioma tissues, respectively, compared to non-tumor brain tissues (NBT). Besides, the expression of BCL7B presented no difference between NBT and glioma (Fig. 1C).

### BCL7s mRNA expression is associated with glioma WHO grade

**Fig. 2:**
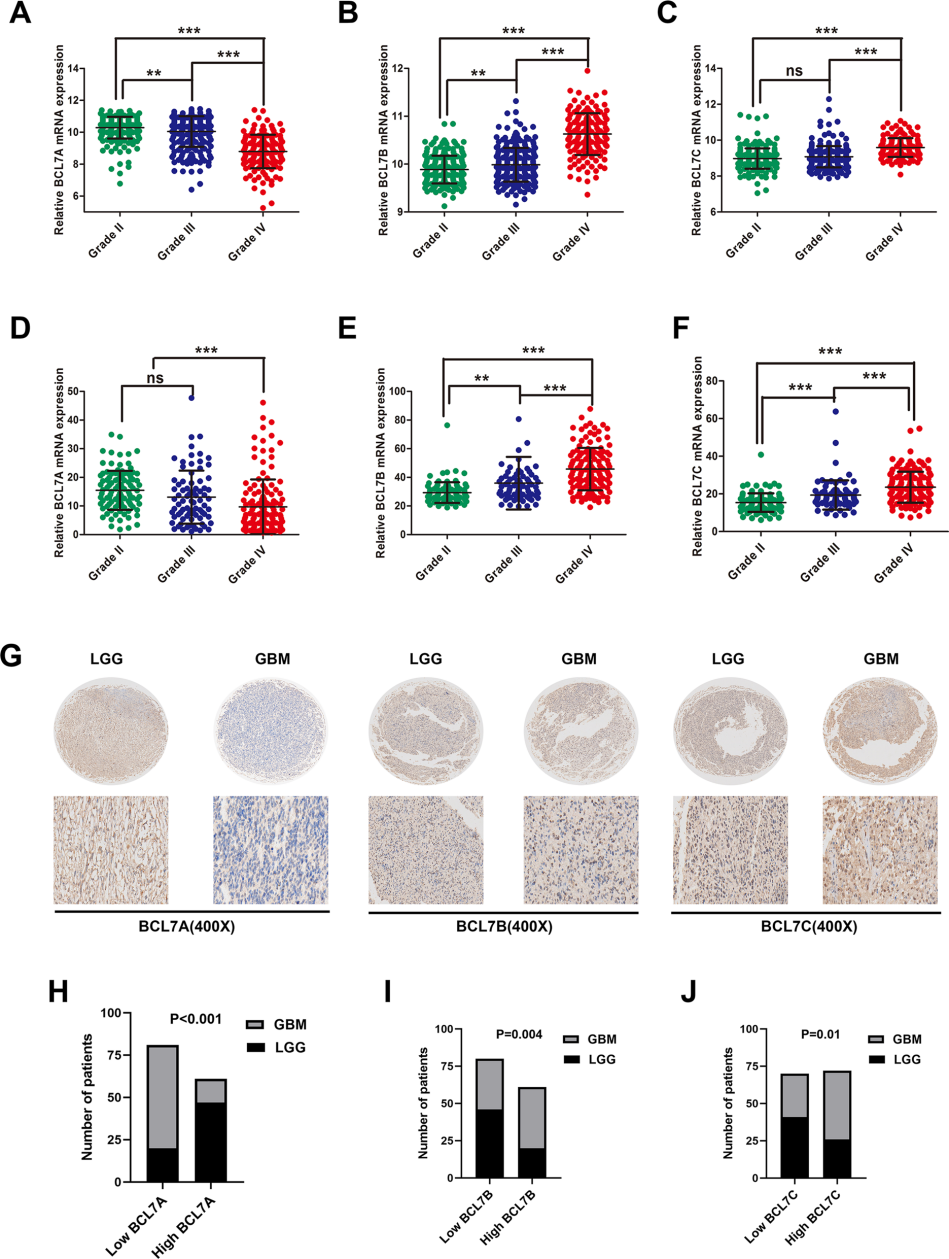
Expression of BCL7 genes associated with glioma WHO grade. Two public datasets, namely TCGA and CCGA were used to explore BCL7A (**A**, **D**), BCL7B (**B**, **E**), BCL7C (**C**, **F**) expression in different grades of gliomas. **, p < 0.01. ***, p < 0.001. ns, no significance. **G** Represented images of IHC staining of BCL7 genes in Lower grade glioma (LGG) and glioblastoma (GBM). **H**–**J** BCL7 genes were differentially expressed between gliomas of different grades

Using two public datasets, (CGGA and TCGA), we evaluated the expression of BCL7s in glioma patients with different tumor grades. Results demonstrated that GBM expressed significantly lower levels of BCL7A mRNA compared to lower-grade glioma (WHO I–III). However, BCL7A expression decreased progressively with higher glioma grade in two public datasets (Fig. 2A, D). Contrarily, BCL7B expression was mainly enriched in GBM and was elevated with an increase in glioma grade (Fig. 2B, E). Also, BCL7C expression was higher in GBM compared to grade II–III glioma tissues in CGGA and TCGA (Fig. 2C, F).

Moreover, we evaluated the expression of BCL7 genes in 108 glioma tissues. Consistent with previous results, BCL7A expression was extremely lower in GBM compared to LGG (Fig. 2G). However, higher levels of BCL7B and BCL7C were reported in GBM than LGG (Fig. 2G). BCL7 genes were differentially expressed among distinct gliomas grades (Fig. 2H–J).

### BCL7A, BCL7B, and BCL7C predict prognosis in glioma

**Fig. 3:**
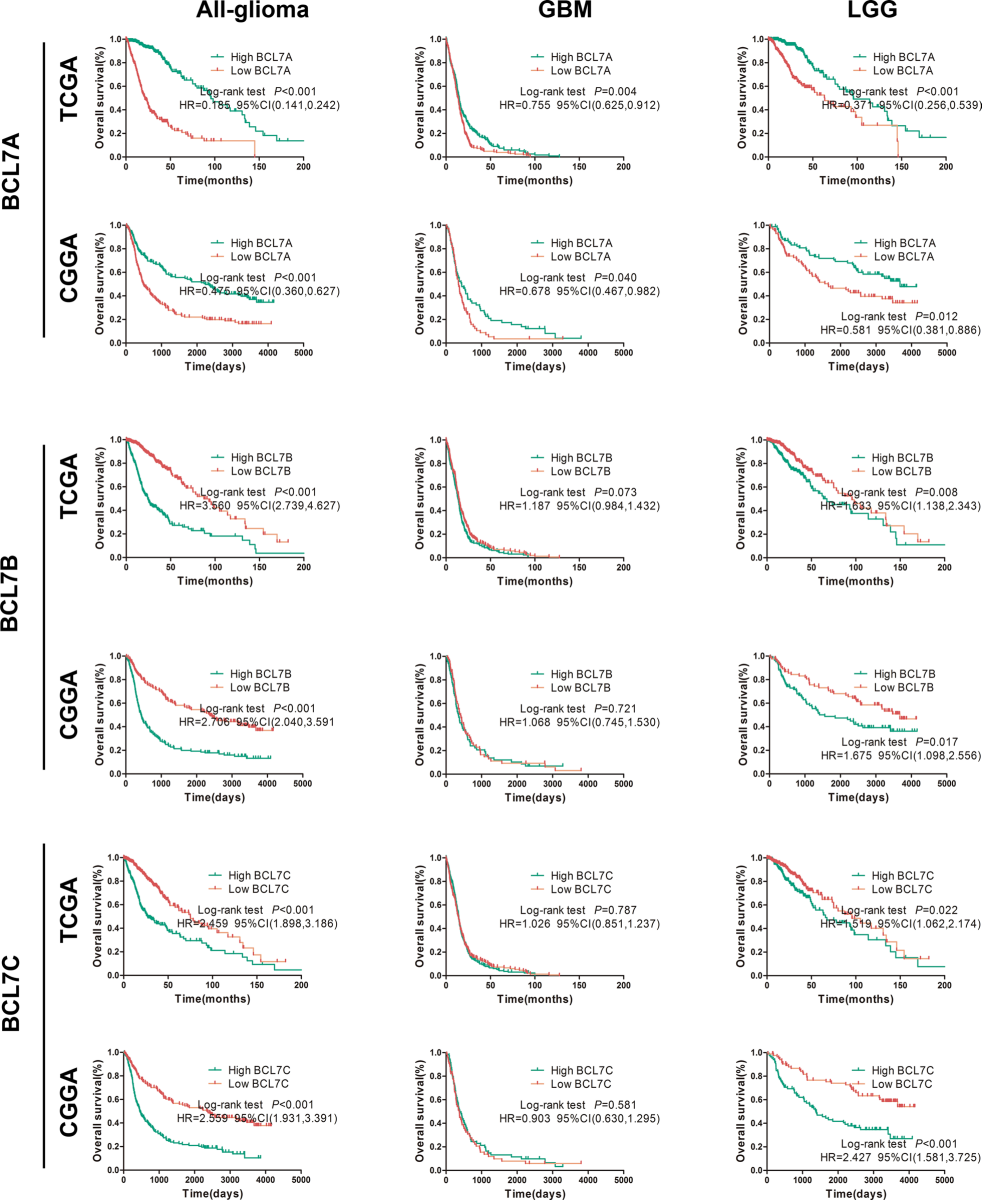
BCL7A, BCL7B and BCL7C predict prognosis in glioma patients. Association between the expression of BCL7s and the overall survival of All-gliomas, LGG (WHO I–III) and GBM (WHO IV) patients were analyzed using Kaplan–Meier method. Median expression value of gene expression was used as the cutoff point. All data were downloaded from Gliovis. HR, hazard ratio

Having strongly demonstrated that BCL7s expression is significantly associated with tumor malignancy, we proceeded to assess their independent prognostic value. Results of survival analysis demonstrated that glioma patients with high BCL7A expression presented a higher percentage of OS than patients with low BCL7A expression in CGGA and TCGA (HR = 0.475 and 0.185, respectively, all p < 0.05, Fig. 3). Besides, the overall survival of glioma patients expressing high BCL7B was significantly worse than their counterparts expressing low BCL7B in CGGA and TCGA (HR = 2.706 and 3.560, respectively, all p < 0.05, Fig. 3). Also, there were consistent trends in the Kaplan–Meier analysis of BCL7C. The OS rate of patients in the high BCL7C expression group was significantly lower than that in the low expression group according to CGGA and TCGA (HR = 2.559 and 2.459, respectively, all p < 0.05, Fig. 3).

The Kaplan‒Meier survival plots depicted the survival of glioma patients with lower-grade glioma (LGG, WHO I–III) and GBM (WHO IV). BCL7A and BCL7B expression could significantly categorize patients with LGG into survival groups on two public datasets (Fig. 3). Low BCL7A expression in GBM patients also predicted poor prognosis in CGGA and TCGA datasets (HR = 0.678, 0.755, respectively, all p < 0.05, Fig. 3). Of note, the effect of BCL7B expression on OS of GBM patients demonstrated no difference in CGGA and TCGA datasets (p > 0.05, Fig. 3). Furthermore, we reported significantly higher overall survival in LGG patients expressing low BCL7C levels than those expressing high BCL7C levels in CGGA and TCGA (HR = 2.427, 1.519, respectively, all p < 0.05, Fig. 6). However, in CGGA and TCGA datasets, BCL7C expression could not classify GBM patients into survival groups (Fig. 3).

### BCL7A acted as an independent risk factor of poor prognosis in glioma patients

Through Multivariable Cox proportional hazard regression analyses, we examined whether BCL7 genes were independently associated with clinical outcome risks. Results showed that high WHO grade (IV), IDH 1/2 wildtype, and low BCL7A expression could be used independently to predict the prognosis of all-gliomas patients based on TCGA and CGGA datasets (Tables 1 and 2, respectively). BCL7B and BCL7C were not independent risk factors for poor prognosis in all-gliomas patients in TCGA and CGGA datasets (Tables 1 and 2, respectively). To further explore the prognostic role of BCL7 genes in GBM patients, we did a multivariable Cox regression analysis. Consistent with previous findings, BCL7A expression in the BCL7 family was solely an independent prognostic indicator of the survival of GBM patients both in TCGA and CGGA datasets (Tables 3 and 4, respectively). Taken together, these findings implicated BCL7A as the only gene in the BCL7 family, significantly associated with the prognosis of all-gliomas and GBM patients. Further, it was argued that BCL7A potentially plays a crucial role in the malignancy process of glioma. However, subsequent studies should mainly focus to elucidate the role of BCL7A in glioma.

**Table 1:** Univariate analysis and multivariate COX analysis of clinical prognostic parameters of all glioma in TCGA dataset

Variables	Univariate Cox regression		Multivariate Cox regression	
	HR (95%CI)	p value	HR (95%CI)	p value
Age	1.08 (1.06–1.12)	< 0.001	1.04 (1.03–1.06)	0.07
Gender (female vs male)	0.89 (0.65–1.22)	0.47	–	–
WHO grade (IV vs I–III)	11.41 (7.68–16.56)	< 0.001	1.98 (1.26–3.10)	0.003
IDH status (wildtype vs mutant)	10.00 (7.05–14.17)		3.82 (2.24–6.51)	< 0.001
BCL7A expression	0.19 (0.13–0.27)	< 0.001	0.61 (0.36–1.04)	0.008
BCL7B expression	3.05 (2.20–4.23)	< 0.001	0.85 (0.55–1.32)	0.47
BCL7C expression	2.62 (1.89–3.62)	< 0.001	1.14 (0.77–1.70)	0.52

**Table 2:** Univariate analysis and multivariate COX analysis of clinical prognostic parameters of all glioma in CGGA dataset

Variables	Univariate Cox regression		Multivariate Cox regression	
	HR (95%CI)	p value	HR (95%CI)	p value
Age	1.03 (1.02–1.04)	< 0.001	1.01 (0.99–1.02)	0.08
Gender (female vs male)	0.97 (0.77–1.21)	0.78	–	–
WHO grade (IV vs I–III)	4.60 (3.63–5.82)	< 0.001	2.95 (2.22–3.93)	< 0.001
IDH status (wildtype vs mutant)	3.50 (2.77–4.42)	< 0.001	2.07 (1.57–2.72)	< 0.001
BCL7A expression	0.57 (0.46–0.72)	< 0.001	0.67 (0.53–0.85)	0.001
BCL7B expression	1.21 (0.97–1.52)	0.09	1.09 (0.85–1.41)	0.49
BCL7C expression	1.34 (1.07–1.68)	0.01	1.23 (0.96–1.59)	0.11

**Table 3:** Univariate analysis and multivariate COX analysis of clinical prognostic parameters of glioblastoma in TCGA dataset

Variables	Univariate Cox regression		Multivariate Cox regression	
	HR (95%CI)	p value	HR (95%CI)	p value
Age	0.93 (0.87–1.04)	0.74	–	–
Gender (female vs male)	0.82 (0.65–1.02)	0.08	1.03 (1.02–1.14)	0.10
IDH status (wildtype vs mutant)	2.90 (1.80–4.69)	< 0.001	2.11 (1.20–3.70)	0.001
BCL7A expression	0.81 (0.71–0.93)	0.002	0.82 (0.65–1.03)	0.03
BCL7B expression	1.28 (1.04–1.58)	0.02	1.16 (0.90–1.50)	0.25
BCL7C expression	0.89 (0.72–1.10)	0.28	0.80 (0.64–1.01)	0.07

**Table 4:** Univariate analysis and multivariate COX analysis of clinical prognostic parameters of glioblastoma in CGGA dataset

Variables	Univariate Cox regression		Multivariate Cox regression	
	HR (95%CI)	p value	HR (95%CI)	p value
Age	1.01 (0.99–1.02)	0.14	1.01 (0.99–1.02)	0.17
Gender Female vs male)	1.03 (0.75–1.40)	0.87	0.96 (0.70–1.32)	0.80
IDH status (wildtype vs mutant)	2.04 (1.32–3.15)	0.001	1.96 (1.26–3.07)	0.003
BCL7A expression	0.61 (0.44–0.85)	0.003	0.66 (0.47–0.94)	0.02
BCL7B expression	1.49 (1.09–2.03)	0.013	1.19 (0.84–1.70)	0.33
BCL7C expression	1.29 (1.04–2.31)	0.013	1.30 (0.92–1.86)	0.14

### Low BCL7A expression associated with IDH wildtype and 1p19q codeletion

**Fig. 4:**
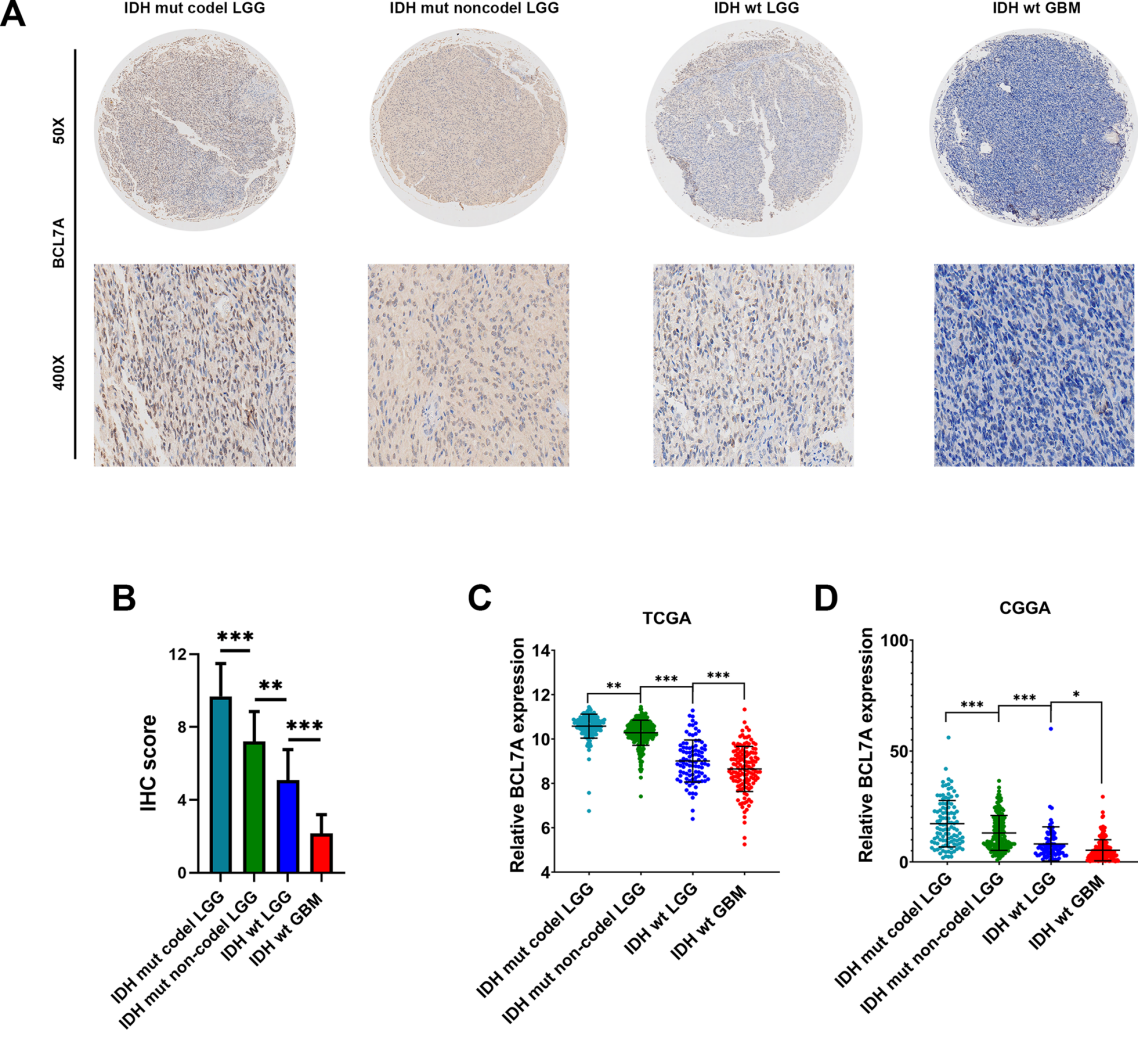
Low BCL7A expression associated with IDH wildtype and 1p19q codeletion. There were 48 LGG tissues and 36 GBM tissues conducted for IDH and 1p19q status testing in our cohort. **A**, **B** BCL7A gene differentially expressed in different IDH and 1p19q status was detected by IHC. Represented images of IHC staining of BCL7A were presented. **C**, **D**. Expression of BCL7A in different IDH1/2 and 1p19q status of glioma in TCGA and CCGA datasets. All data were downloaded from Gliovis. IDH, isocitrate dehydrogenase. WT, wildtype. Mut, mutant. *, p < 0.05. **, p < 0.01. ***, p < 0.001. ns, no significance

IDH1/2 mutations and 1p19q co-deletion, the 2 crucial mutations in glioma, are associated with favorable prognosis in glioma patients. The 2 mutation test has become a part of the routine diagnosis and classification of gliomas [18, 19]. Herein, LGG patients were classified into three groups (Group 1: IDH1 wild-type, Group 2: IDH1 mutations with 1p19q codeletion, and Group 3: IDH1 mutations without 1p19q codeletion) according to the guideline of the 2016 WHO classification of CNS tumors. Through immunohistochemistry staining, we found significantly lower BCL7A levels in IDH1/2 wildtype GBM than in LGG (Fig. 4A). Also, LGG patients with IDH1 mutations and without 1p19q codeletion (Group 3) exhibited higher BCL7A expression than patients with IDH1 wild-type (Group 1) or those with IDH1 mutations and 1p19q codeletion (Group2) (Fig. 4A, B).

To validate these findings, further analysis was implemented using data from CGGA and TCGA. We revealed that LGG patients in Group 3 (IDH1 mutations without 1p19q codeletion) highly expressed BCL7A than patients in Group1 and Group 2 (Fig. 4C).

### BCL7A is lowly expressed in mesenchymal GBM

**Fig. 5:**
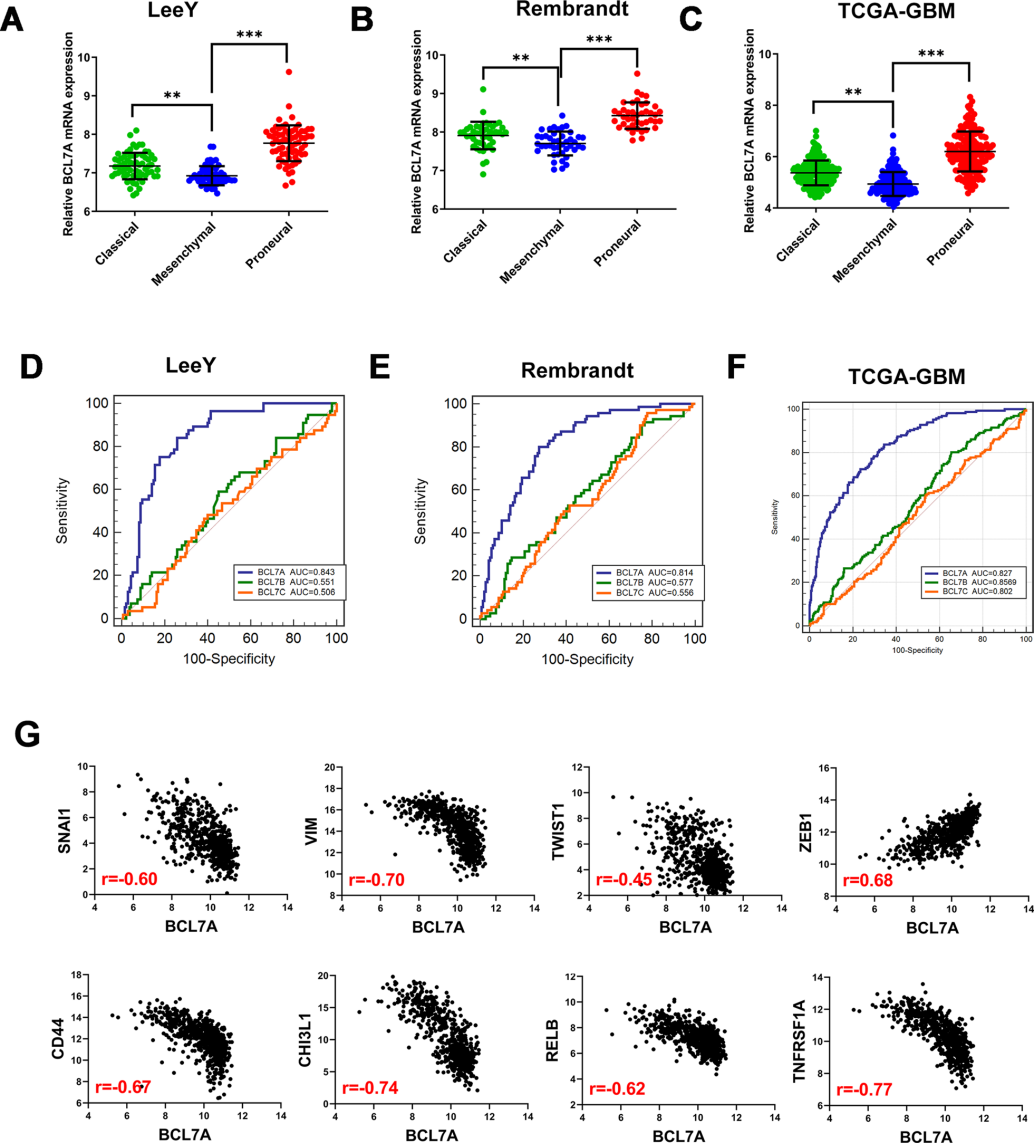
BCL7A expression enriched in mesenchymal GBM. **A**–**C** Three public datasets, namely LeeY (GSE13041), Rembrandt and TCGA-GBM were used to explore BCL7A expression in different molecular subtypes of GBM. **D**–**F** ROC curves of BCL7 genes in predicting mesenchymal subtype in GBM in LeeY (GSE13041), Rembrandt and TCGA-GBM datasets. **G** Correlation between BCL7A and mesenchymal-related genes TCGA datasets. Spearman correlation analysis was employed. All data were downloaded from Gliovis. AUC, area under curve. *, p < 0.05. **, p < 0.01. ***, p < 0.001. ns, no significance

Previously, Verhaak et al. subclassified GBM into four subtypes (classic, mesenchymal, proneural, and neural) based on similarity to defined genomic expression signature [3]. GBM cells with a mesenchymal phenotype, in contrast to epithelial tumor cells, showed higher expression levels of cell movement-associated proteins in addition to improved migration and invasion [20, 21]. Herein, BCL7A expression in LeeY (GSE13041), Rembrandt, and TCGA-GBM (Hg-U133) molecular subtypes of GBM was evaluated. We found a lower expression of BCL7A in mesenchymal subtype GBM than in other subtypes (Fig. 5A–C). To demonstrate the predictive power of BCL7A on the mesenchymal phenotype, a Receiver Operating Curve (ROC) analysis was undertaken. The potential of BCL7A to predict the mesenchymal subtype of GBM was more powerful than BCL7B/C. The Area Under Curve (AUC) was 0.843, 0.814, 0.827 for BCL7A in LeeY, Rembrandt, and TCGA, respectively (Fig. 5D–F).

By estimating the expression correlation coefficient between BCL7A and mesenchymal-related genes using the Spearman Correlation method, we found a significant correlation of BCL7A with multiple epithelial-mesenchymal transition (EMT) related markers (Snail1, Vimentin, Twist1, and ZEB1), and mesenchymal-related genes (CD44, CHI3L1, RELB, and TNFRSF1A) (Fig. 4E). Collectively, these findings implied that BCL7A is enriched in the mesenchymal subtype, thus, we hypothesized that BCL7A could influence the change from an epithelial to a mesenchymal phenotype.

### BCL7A is associated with immune cell infiltration in glioma

**Fig. 6:**
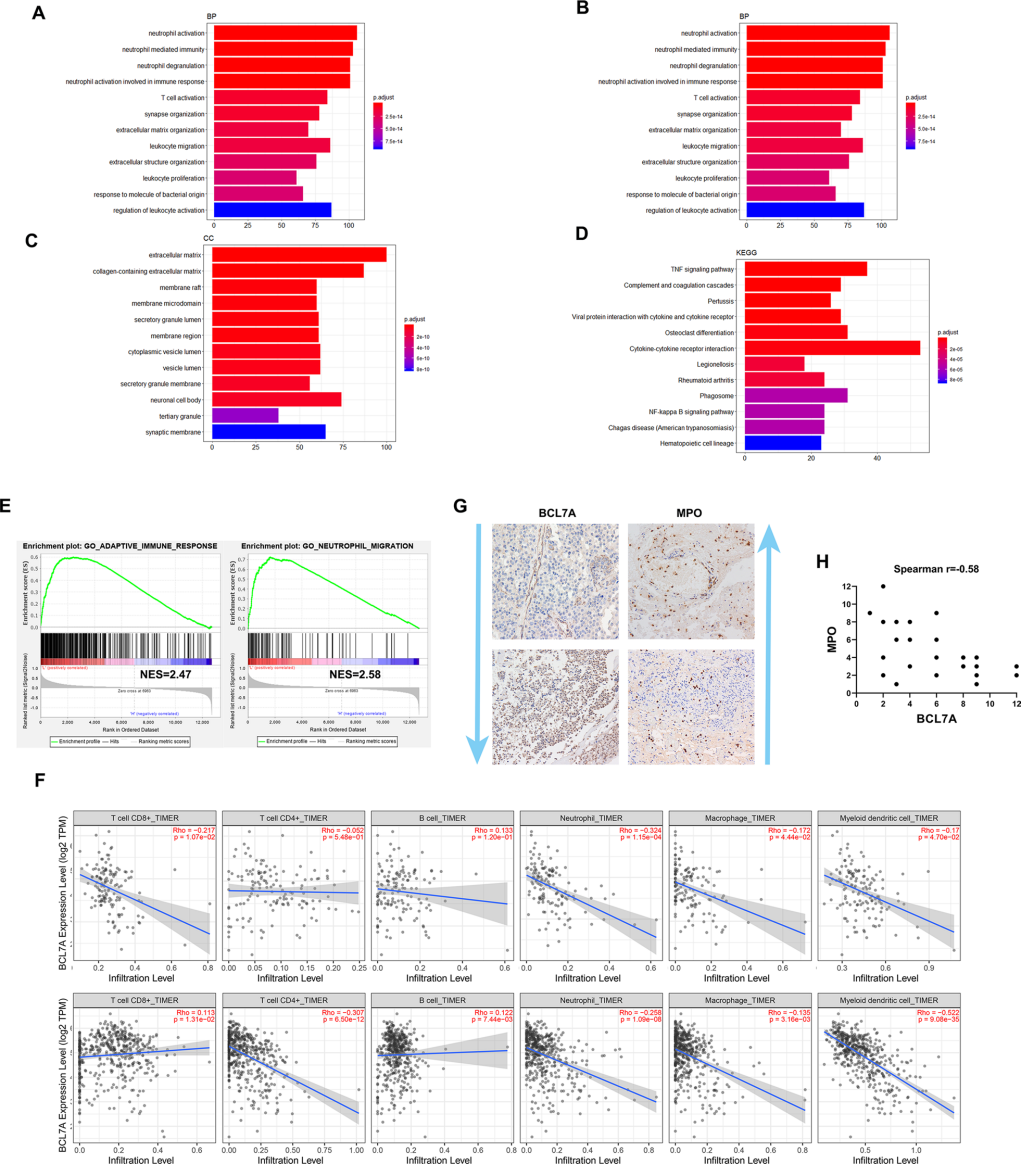
BCL7A associated with immune cell infiltration in glioma. GO enrichment analysis significantly distributed DEGs into biological process (**A**), molecular function (**B**), cellular component categories (**C**) and KEGG pathways prediction (**D**). **E** Gene-set enrichment analysis (GSEA) of BCL7A in GBM. NES, normalized Enrichment score. **F** Correlation between BCL7A and immune cell infiltration in LGG and GBM according to TIMER. **G** IHC staining was employed to detect BCL7A expression and MPO in glioma tissues. **H** Correlation between the BCL7A and MPO immunohistochemistry (IHC) score. Spearman correlation analysis was employed

GO enrichment analysis significantly distributed DEGs into biological process, molecular function, and cellular component categories. Consequently, BLC7A was mainly enriched in neutrophil activation (GO:0042119), extracellular matrix structural constituent (GO:0005201), cell adhesion molecule binding (GO:0050839), extracellular matrix (GO:0031012), and collagen-containing extracellular matrix (GO:0062023) (Fig. 6A–D). Following gene-set enrichment analysis (GSEA) of BCL7A in GBM, the adaptive immune response and neutrophil migration were highly enriched in BCL7A low expression group (Fig. 6E). TIMER was applied to explore the correlation of BCL7A with immune cell infiltration and reported a significant negative-correlation between BCL7A expression in tumor cells and the infiltration of neutrophil cells in LGG and GBM (Fig. 6F). IHC analysis revealed robust infiltration of neutrophil in the glioma tissues of low BCL7A expression; however, less infiltration of neutrophil occurred in tissues of high BCL7A expression (Fig. 6G, H).

### BCL7A is an independent predictor of response to TMZ in LGG and GBM

**Fig. 7:**
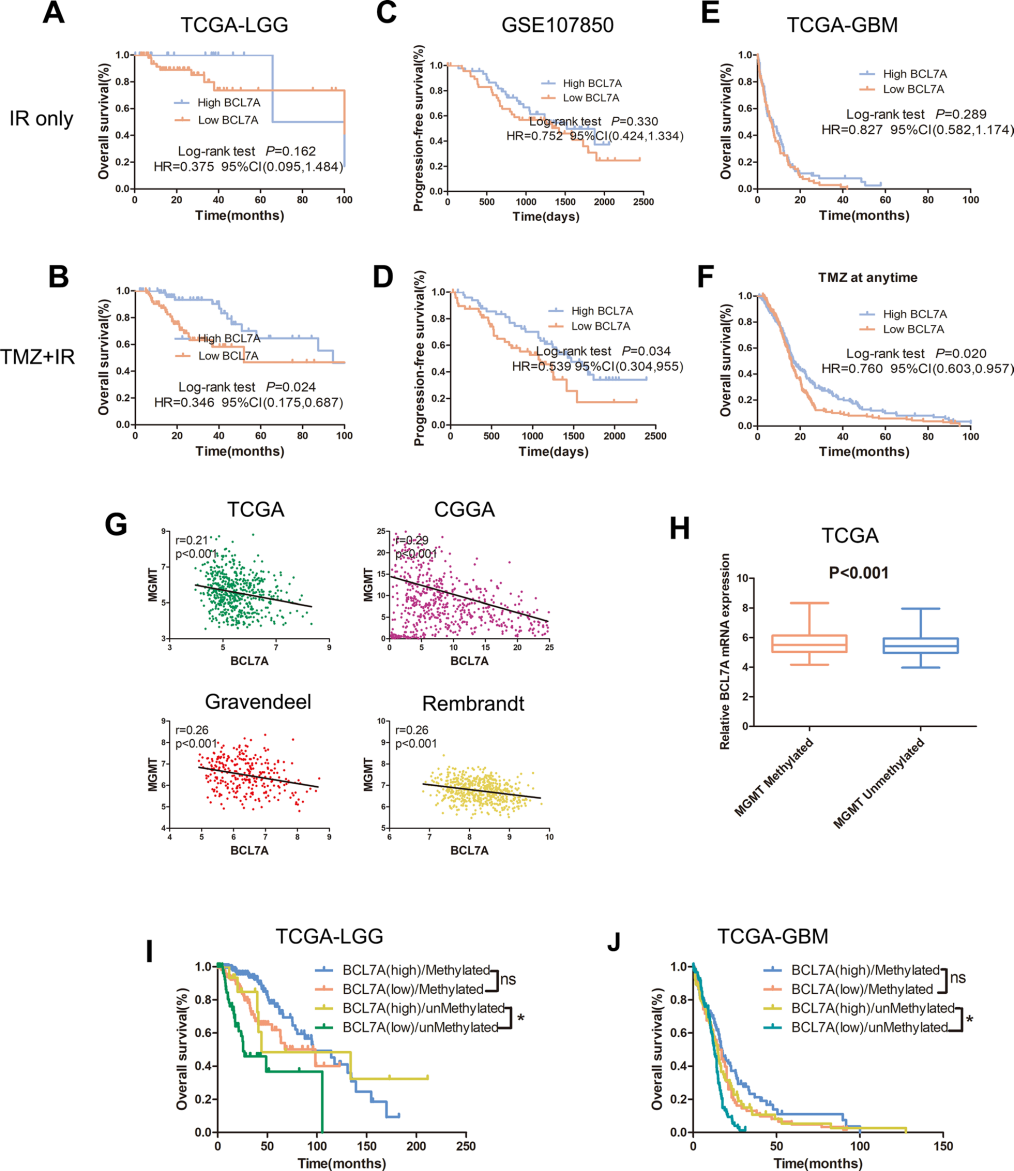
BCL7A is an independent predictor of response to TMZ in glioma. **A**, **B** Kaplan‒Meier survival plots depicting survival of (IR only)-treated or (TMZ + IR)-treated LGG patients with different BCL7A expression in TCGA-LGG. **C**, **D** GSE107850 was used to verify the association between BCL7A and prognosis of LGG patients with different treatments. **E**, **F** Effect of BCL7A on prognosis of GBM patients treated with IR or TMZ + IR. **G** Correlation between BCL7A and MGMT in GBM in four public datasets. **H** Expression of BCL7A in patients with different MGMT promoter methylation. **I**, **J** Effect of BCL7A on prognosis of LGG and GBM patients separated by MGMT promoter methylation status. HR, hazard ratio. IR, ion radiotherapy. *, p < 0.05. ns, no significance

Since BCL7A expression is highly enriched in mesenchymal GBM and strongly associated with immune response in glioma, we attempted to explore whether BCL7A functioned as a predictive biomarker for positive adjuvant chemotherapy response. Patients were categorized into BCL7A high andBCL7A low groups, based on the mean expression levels of BCL7A in all samples. Results in TCGA-LGG showed that patients receiving ion radiotherapy (IR) only in the high BCL7A group had no difference in prognosis compared to those in the low BCL7A group (p > 0.05, Fig. 7A). Besides, patients who received chemotherapy (TMZ) combined with IR in the high BCL7A group survived longer than patients in the low BCL7A group (HR = 0.346, 95% CI [0.175, 0.687], p < 0.05, Fig. 7B). Similar results were reported in GSE107850, a dataset we adopted to explore whether TMZ confers a survival advantage over IR in LGG (Fig. 7C, D). Importantly, there was no effect of BCL7A expression on the overall survival of GBM patients who received IR only (p > 0.05, Fig. 7E). However, BCL7A separated GBM patients who received TMZ + IR into survival groups (HR = 0.760, 95% CI [0.603, 0.957], p < 0.05, Fig. 7F). These findings demonstrated the potential influence of BCL7A on the effect of TMZ in glioma. Considering the association of MGMT promoter methylation status with TMZ resistance, we explored the relationship between BCL7A and MGMT. Notably, BCL7A expression was negatively associated with MGMT expression in four public datasets (Fig. 7G); its expression was highly enriched in MGMT methylated glioma (Fig. 7H). In LGG and GBM patients with MGMT methylation, BCL7A expression could not segregate GBM patients into survival groups (Fig. 7I, J). Surprisingly, patients with unmethylated MGMT promoter in the high BCL7A group presented a higher percentage of overall survival than patients in the BCL7A low group (Fig. 7J). LGG and GBM patients with methylated MGMT in the low BCL7A group presented a similar prognosis to patients with unmethylated MGMT in the high BCL7A group.

## Discussion

There is evidence from recent studies on the association of the BCL7 family with tumorigenesis and progression of cancers [12, 22, 23]. However, expression and the biological function of BCL7 genes in glioma had not been explored. In this study, we report that BCL7A significantly is associated with tumor malignancy. Although BCL7A appeared less enriched in mesenchymal GBM, it may function as a negative regulator responsible for the change from an epithelial to a mesenchymal phenotype. Further analysis revealed that patients who received chemotherapy (TMZ) combined with IR with high BCL7A expression survived longer than patients with low BCL7A expression. Thus, the present study strongly implicates BCL7A as a novel tumor suppressor gene, pivotal for predicting the response to TMZ in glioma.

BCL7 genes are specific subunits of the SWI/SNF chromatin-remodeling complex. Although expression of BCL7C in the brain is unclear, BCL7A and BCL7B are highly expressed throughout the brain, including the cortex and hippocampus. Knocking out BCL7A in mice brain reduces dendritic arbors of Purkinje cells, subsequently impairing animal survival and behavior. Besides, BCL7B knockout did not affect Purkinje cells [5]. BCL7A and BCL7B were identified as the two of the lowly expressed candidate genes associated with a small region of loss at 10q26.3 and 7q11.23 in astrocytoma, respectively [24]. Whilst acknowledging the wide studies of BCL7A in multiple lymphoid neoplasms, including Hodgkin lymphoma, T cell lymphoma, and Burkitt lymphoma, it was less explored in solid tumors. Also, the involvement of BCL7A locus in a recurrent breakpoint in lymphomas and MYC-BCL7A fusion transcript in Wien 133 cells had earlier been demonstrated [8]. By screening differential methylation between 28 patients with primary cutaneous T-cell lymphoma (CTCL) and benign T-cell samples, BCL7A demonstrated a higher frequency of hypermethylated. The above findings inferred BCL7A as a putative tumor suppressor gene [10]. Moreover, another exploration of BCL7A mRNA expression showed that it was enriched in stage I CTCL. Less BCL7A expression is associated with a more favorable prognosis of CTCL patients [25]. Herein, we demonstrated the BCL7A expression pattern in glioma for the first time. Significantly lower BCL7A expression was found in glioma tissues compared to non-tumor brain tissues. Furthermore, BCL7A expression was negatively correlated with glioma grades. These results strongly suggest that BCL7A is a potential novel tumor suppressor gene in glioma, similar findings were reported previously [26]. We also investigated the prognostic role of BCL7A for the first time in glioma. Elsewhere, ovarian cancer (OC) patients with high expression of BCL7A had shorter overall survival (OS) /progression-free survival (PFS) than those with low levels of BCL7A. BCL7A expression was implicated as an independent risk factor for poor prognosis OC patients [26]. Consistent with the role of BCL7A in OC, we identified BCL7A as a novel prognostic biomarker for LGG patients and GBM patients. BCL7A was the only gene among the BCL7 family that independently was associated with the prognosis of glioma patients.

Recently, researchers have yielded exciting findings in the field of tumor metabolism. Some aspects by which these metabolic alterations are related to IDH mutations have also been described. IDH1 mutant cells have metabolic profiles, distinct from those of IDH wildtype cells [27]. The discovery of neomorphic 2HG production as a consequence of recurrent IDH 1/2 mutations in glioma and other malignancies has strengthened the proposed link between abnormal cellular metabolism and epithelial-mesenchymal transition (EMT). Low expression of BCL7A in IDH wildtype and mesenchymal glioma tissues indicates that it potentially mediates the IDH-induced EMT. This hypothesis was also supported by a significant association between BCL7A and multiply EMT-related markers. Elsewhere, scholars found that mutant IDH-induced EMT was dependent on the up-regulation of the transcription factor ZEB1 and down-regulation of the miR-200 family of microRNAs [28]. It is reasonable to assume that BCL7A could be a crucial molecular that related to EMT process, although this needs further validation of molecular biology experiments.

Another study uncovered a close association of BCL7A with immune cell infiltration and chemoresistance, although the precise mechanism of interaction remains unknown. Numerous studies also demonstrated that the administration of TMZ could either positively or negatively influence the dynamics of the tumor microenvironment (TME) by altering immune cell infiltration or components of the TME [29–31]. This might be a potential mechanism underlying BCL7A-associated chemoresistance. We found that BCL7A mediated TMZ modulated-TME. It could regulate the infiltration of immune cells via a different mechanism, for example, as an EMT regulator. The possible reason might be that BCL7A-regulated precancerous cells in the tumor microenvironment undergo EMT and then acquire the phenotype of infiltration and chemoresistance [32]. In a previous study, the epidermal growth factor (EGF) receptor partly mediated TMZ resistance in GBM, suggesting the potential immune therapy with EGF as the target [33]. The regulatory relationship among BCL7A, EMT process, and immune modulation could be more appropriate to the mechanism underlying BCL7A mediating chemoresistance.

There were some shortcomings in this study. The glioma tissue microarray used contained 108 glioma samples, whereas only a subset of patients’ prognosis was evaluated during the follow-up. Thus, Univariate and multivariate COX analyses need further validation. Besides, the mechanism by which BCL7A influences glioma cells in vitro remains unclear, which should be established through molecular biology experiments. Further studies may elucidate the potential mechanisms that underlie the progression of glioma and provide a novel molecular therapeutic target for its management.

## Conclusion

The present study implicates BCL7A as a new tumor suppressor gene, negatively associated with glioma malignancy. It can be utilized as an independent prognostic biomarker and represents a novel predictor of response to TMZ in glioma.

## Data Availability

The data that support the findings of this study are available from the corresponding author upon reasonable request.
